# Shedding light on autistic traits in struggling learners: A blind spot in medical education

**DOI:** 10.1007/s40037-021-00654-z

**Published:** 2021-02-20

**Authors:** Marie Giroux, Luce Pélissier-Simard

**Affiliations:** 1 Department of Family Medicine and Emergency Medicine, Université de Sherbrooke, Sherbrooke, Canada; 2 Professional Development, Pedagogy and Social Accountability, Université de Sherbrooke, Sherbrooke, Canada

**Keywords:** Autistic traits, Neurodiversity, Medical education, Competency-based medical education

## Abstract

Some highly challenging, seemingly “unsolvable” situations that arise in medical education could be the result of autistic traits (AT) in learners. AT exist in physicians and learners, ranging from profiles compatible with DSM-5’s criteria for autism spectrum disorder (ASD) to more subtle manifestations of ASD’s “broader phenotype.” Often associated with strengths and talents, AT may nonetheless pose significant challenges for learning, teaching, and practising medicine. Since AT remain widely under-recognized and misunderstood by educators, clinicians, and affected individuals alike, they represent a blind spot in medical education. The use of a “neurodiversity lens” to examine challenging situations may help educators consider different pedagogical approaches to address those potentially stemming from AT.

This paper aims to raise awareness and understanding of AT-related difficulties in struggling medical learners. To overcome the blind spot challenge and help develop this “neurodiversity lens,” we explore different angles. Beyond any diagnostic consideration, we offer a series of contextual examples, paralleled with explanatory concepts from the field of ASD. We also underline the role of context on functional impact and describe the often ill-defined pattern of challenges encountered, as well as the fertile grounds for interpersonal misunderstandings and disrespect. We propose historical, cultural, and clinical reasons likely contributing to the blind spot. Mindful of the potential risks of prejudice associated with identifying AT-related difficulties, we underline the necessity and feasibility of conciliating diversity and dignity with accountability standards for medical competence.

## Introduction

Certain challenging interpersonal situations encountered in medical education leave both learner and teacher frustrated and with a feeling of unfinished business. We can all remember learners who have repeatedly made “surprising” or inappropriate decisions, seemingly unaware of the cues expressed by others and unable to understand their role in creating the problematic situation. Often interpreted as deficiencies in communication skills or professionalism, these situations can feel “unsolvable” to medical educators.

However, when identifying struggling learners’ deficits, medical educators generally overlook cognitive characteristics related to autistic traits (AT). Individuals with AT process information differently and usual remediation strategies may not work. Because of their potential impact on learning, communication, and interactions, AT deserve to be better understood by medical educators and the entire academic medical community.

Despite increasing literature regarding students with autism in higher education, very little has been published on AT as they relate to medical education. A search of the gray literature and databases in medicine, education, and psychology using relevant keywords revealed only one research article [1], four comments [2–5] and one book chapter [6] on this subject, pointing to a blind spot in medical education.

This paper aims to help medical educators identify neurocognitive characteristics in struggling learners that, if unrecognized, are likely to affect the achievement of expected competences. The first step is to acknowledge individual differences in cognitive processes and understand how AT might explain learning challenges. This possibility broadens the scope of hypotheses for performance gaps, may dampen educators’ negative assumptions, and enable comprehensive approaches to support optimal learning.

We hope to begin a necessary conversation in medical education about this delicate, ever-evolving [7], and potentially controversial topic [8, 9]. AT can present as differences, difficulties, deficits and/or as strengths and talents depending on context, expectations, and perspectives [9]. Recognizing AT is a non-issue when learners meet expectations. However, if challenges arise, a “neurodiversity lens” may prevent an escalation of problematic behaviours as described below.

A full review of autism in medical learners is beyond the scope of this paper. Most autism-related theories and paradigms, including those highlighted in this paper, are controversial within both the autism and the scientific communities. Our aim is simply to help medical educators consider viewing puzzling situations through a new lens.

## Autistic traits within the neurodiversity continuum

The use of various terms deserves clarification. We focus here on neurocognitive characteristics termed “autistic traits” (AT), rather than on a specific diagnosis like autism spectrum disorder (ASD). By AT, we mean a combination of features associated with ASD, which is characterized by persistent deficits in social communication and restricted, repetitive patterns of interest [10]. Below diagnostic thresholds for ASD, AT extend into a “broader phenotype” with similar characteristics but less functional impact [11–13]. Beyond any diagnostic consideration, information processing is atypical to varying degrees [11].

Neurodiversity is a paradigm that aims to de-medicalize neurocognitive “disorders” and promotes respect and inclusivity for individual differences [9, 14–17]. Paralleled with biodiversity, it originated from the autism community and now includes other developmental conditions, such as attention-deficit hyperactivity disorder (ADHD), dyspraxia, Tourette’s syndrome, and intellectual disability. Clinically, ASD and its “broader phenotype” reveal a broad range of phenotypes, include many overlapping conditions, and suggest a continuum in traits and disability in which “neuroatypicality” blends with “neurotypicality.”

Here, we position AT as an expression of neurodiversity and a specific manifestation of neuroatypicality. We believe that AT deserve specific attention which cannot be meaningfully addressed through “neuroatypicality” in its broader sense. Indeed, AT carry singular social communication difficulties which may explain distinctive challenges and are particularly poorly recognized in medical education. A “neurodiversity lens” offers a non-stigmatizing potential explanation for challenging interpersonal situations, acknowledging that these difficulties may impact learning and competency. It recognizes AT’s frequent distinctive features, described below.

The term “high-functioning autism,” broadly used in autism literature [18, 19], represents a particular expression of AT at the ASD pole of the neurodiversity continuum. It encompasses the set of atypical cognitions, attitudes, and behaviours in individuals with normal or above average IQ and good potential for functional compensation of underlying difficulties. This term generally includes Asperger syndrome, embedded in ASD in the DSM‑5 [10]. As such, it appears a relevant reference for learners who have entered medical school.

## Autistic traits in medical learners

Although the prevalence of ASD within the medical community is unknown, AT are increasingly recognized in this group. Discussions with colleagues from human resources, physician health services, and among peers are consistent: some medical learners, practising physicians, and faculty are likely on the spectrum or close to it.

### Contextual examples

**Fig. 1:**
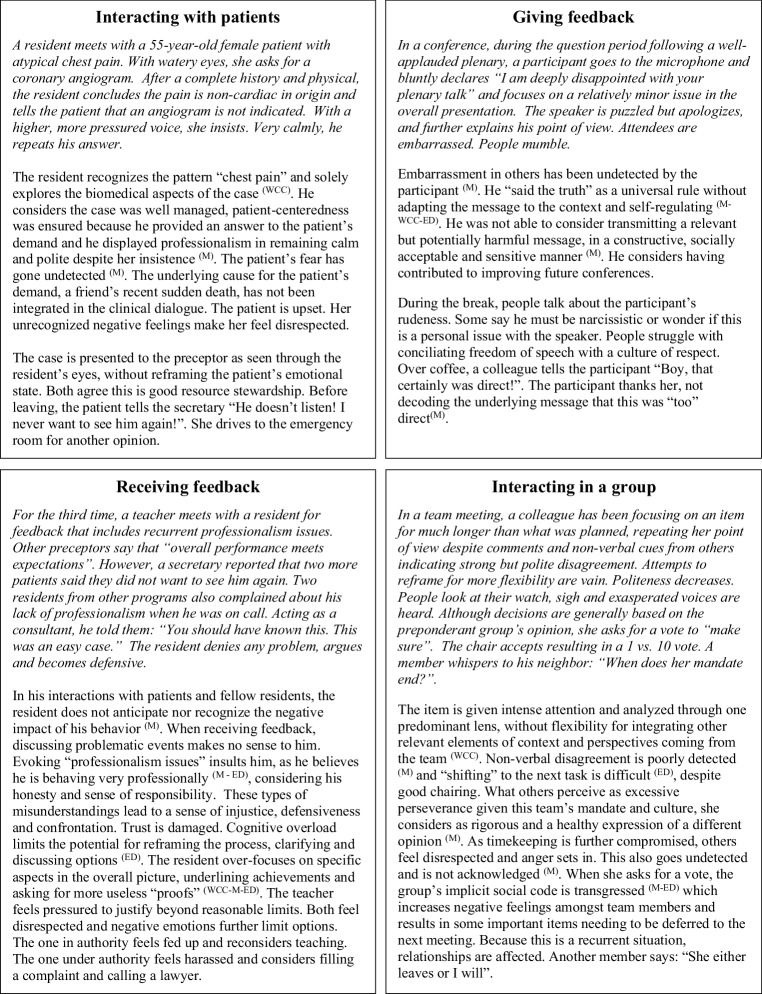
Contextual examples of AT-related difficulties with compounding effect of Mindblindness ^(M)^, Weak Central Coherence ^(WCC)^ and/or Executive Dysfunction ^(ED)^

While the stereotyped severe clinical presentations of ASD are generally clear, more subtle AT may be difficult to recognize in high-performing individuals such as practitioners and medical learners. Fig. 1 presents individuals displaying AT rarely recognized as such without using a neurodiversity lens. Each example refers to AT-related characteristics facilitating their recognition in learners with problematic communication, information processing, and behaviour.

### Schematic representation of AT-related characteristics

**Fig. 2:**
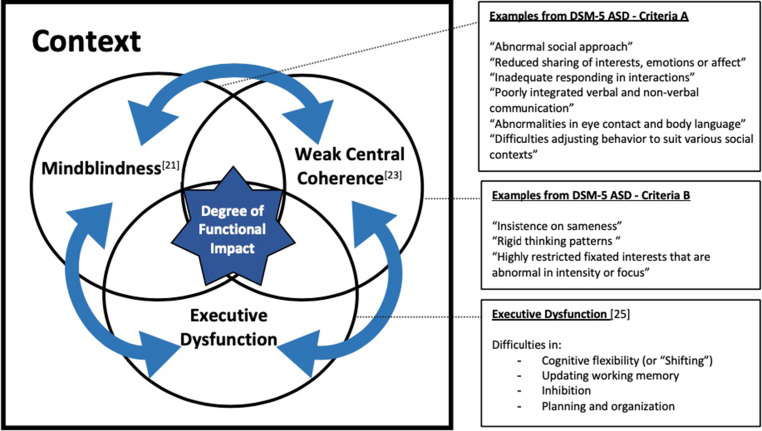
Schematic representation integrating three important neurocognitive characteristics related to Autism Spectrum Disorder (ASD) with links to examples from the DSM‑5 [10] diagnostic criteria that may apply to medical learners with Autistic Traits (AT)

Fig. 2 presents the overlap and interaction of three integrated sets of AT-related characteristics described in the autism literature, including Attwood’s [20] comprehensive work on Asperger syndrome.

First, *mindblindness* or *Theory of Mind* by Baron-Cohen [21] refers to a limited capacity for “reading” what other people think and feel, “putting oneself in someone else’s shoes,” anticipating reactions, and “reading between the lines.” Difficulties in interpreting verbal and non-verbal cues in interactions limit resonance, empathy, and emotional self-regulation, often leaving others with an impression of flawed communication. Natural interpersonal skills, e.g., integrating implicit social codes and understanding someone’s state of mind solely from their facial expressions [22], are challenging for people with AT. Lack of socio-emotional reciprocity generally dominates interpersonal exchanges, and a literal communication style prevails. Decoding emotions in self or others is challenging and emotional information readily results in cognitive overload. Abstraction is limited and understanding of metaphors is arduous [20].

Second, *weak central coherence*, described by Frith and Happé [23], involves difficulty seeing the “big picture” of a given situation. Attwood [20] compares it to looking at situations through a cylinder. As attention readily focuses on details, specific parts of a situation are over-invested while the context is missed. Rules and facts dominate over common sense. Things are approached one at a time through one predominant lens. Cognitive patterns are rigid. Attention is drawn to anomalies in familiar patterns and to negative aspects of situations.

Third, *executive dysfunction*, related to Theory of Mind by Ozonoff et al. [24], occurs frequently in ASD as well as depression and ADHD. Executive functions are high-level cerebral processes coordinating other cognitive functions, allowing timeliness of appropriate strategies and relevant adaptation to new information [25]. Among them, “shifting” (cognitive flexibility) enables swift adaptation of personal behaviour to changes in the environment. “Inhibition” (impulse control) controls inappropriate or disruptive reactions. “Updating” allows regular integration of incoming information into working memory, keeping it readily available for subsequent thinking processes. “Planning/organization” enables setting priorities and spending time and energy on the right tasks, in a timely and efficient manner. These processes are cornerstones for learning, metacognition, self-reflection, self-regulation, and adaptiveness. The abundant evidence published on these subjects in the psychology and education literature further supports the notion of neurodiversity and highlights the potential challenges related to AT [8, 9, 26].

None of these three characteristics is specific to AT as they can occur sporadically in anyone. Fig. 1 echoes the schematic representation in Fig. 2. Heterogeneity prevails. In terms of functional impact, context is key and moving thresholds for acknowledging problems is hallmark. At the more atypical pole of the continuum, ASD includes “deficits across multiple contexts” and “clinically significant impairment” [10]. In different contexts and from different persons’ perspectives, the same AT may range from subclinical, sometimes even considered as strengths, to a significant dysfunction. Differences may never become deficits in contexts requiring little interaction or need for adaptation and learning. Deficits may be minimized for years and be considered as one’s “norm” as AT remain unrecognized despite evident impairment in interactions.

The strengths associated with AT can be remarkable and may explain highly valuable professional achievement. In some contexts, individuals have either compensated for AT-related challenges or have honed AT into expressions of excellence. Excellent systematizing skills and pattern recognition [27] as well as exceptional visual and long-term memory are common in this group, as are a high moral sense, honesty, and a strong sense of social justice [20]. To overcome communication challenges, some individuals with AT break typically intuitive interpersonal skills into a series of explicit steps, potentially making them excellent educators. Persistent and intense focus on a specific subject, with relentless attention to details, leads some to widespread recognition as experts.

### Patterns of challenges

While AT are not linked to a specific and consistent behaviour and often go unnoticed, some patterns in challenges suggest their presence in struggling learners. When learning issues occur, they are usually non-specific, vague, heterogeneous, and include communication or professionalism issues. They can involve knowledge, attitudes, behaviours, and skills in different rotations or professional settings, although not necessarily all of them nor with the same intensity (“inconsistent performance”). Progression often proves uneven, depending on the individual’s capacity to meet expectations and cope with stress in each context (“he/she had a similar difficulty before, but things had gotten better”). Changes in context or increasingly complex responsibilities may be associated with a plateau or decreasing performance. Difficulties may appear in contexts requiring refined reading of patients’ or team members’ emotions and non-verbal cues.

Significant interpersonal challenges may occur, generally witnessed by different sources over time and labelled as professionalism issues. Persistent difficulties in decoding the social components of situations, with limitations in recognizing impacts on others and providing adequate response, can foster negative interactions with patients, teachers, other learners, colleagues, and staff. People may express feeling disrespected. They occasionally evoke ADHD if executive dysfunction is significant, label apparent coldness as narcissism, or conclude rudeness if poor anger management occurs.

Examples in Fig. 1 illustrate how the interactions of mindblindness, weak central coherence, and executive dysfunction can lead to highly varied, complex, and escalating situations. Mindblindness and executive dysfunction may compromise appropriate social filters in communication, behaviour, and attitude leading to interactions that are problematic in content, tone, and intensity. A genuine intention to “help others improve” or “aim for a greater good,” expressed by an individual with AT in what they consider “straight talk” or “healthy conflict,” may be interpreted by others as poor empathy. Imbalanced socio-emotional reciprocity can translate into talking too little or too much, to the point of being overly motivated or inappropriately intrusive with others [20]. Mindblindness further limits capability to integrate external cues to downregulate.

With weak central coherence, over-focusing on specific aspects of a given task or situation prevents seeing “the big picture.” Added to executive dysfunction, time management, setting priorities, reasoning, organization of knowledge, and overall judgment may become problematic. In clinical reasoning, premature closing of hypotheses occurs when clinical patterns are “recognized” and adopted too soon. Mental schemas may be poorer than expected or patchy if the learner over-invests specific areas of interest while neglecting other important concepts. Limits in insight and impulse control might result in clinical overconfidence, along with difficulties in anticipating consequences for patients. Comprehensiveness and patient-centeredness often prove challenging, as the patient’s context, emotions, expectations, and preferences are not well understood.

Difficulties in social communication can lead to misunderstandings escalating into disrespectful behaviours. An individual with AT is often unaware of personal limitations apparent to others and may not accurately gauge the intensity of non-verbal reactions nor adjust appropriately. Others, including patients, may interpret this as “adding insult to injury.” If misunderstandings build up, emotional reactions can grow stronger, and at one point, the individual with AT may become upset with the “sudden” intensity of another person’s reaction. In teamwork, like in rounds or meetings, not understanding and adjusting to implicit group norms or non-verbal cues can affect the group’s performance and damage relationships.

When receiving negative feedback, there may be a limited capacity to understand what is said or to acknowledge difficulties and engage in a constructive dialogue. Even legitimate feedback might generate a sense of injustice. Misunderstandings build up, a confrontation ensues, and trust is damaged, further limiting options. If recurrent, the learner may wrongfully infer harassment and file a complaint or engage in a legal process.

## The blind spot

Many clinical, historical, and cultural factors, including some individual and systemic cognitive biases curtail the recognition and understanding of AT within the medical community. Atypical information processing and difficulties in social communication can be quite counterintuitive for neurotypical individuals. Challenges in interpersonal relationships seem to be generally interpreted through a “willingness” lens rather than a “capability” (neurological) lens.

Clinical experiences influence medical educators who may see ASD mostly as a childhood issue and an all-or-none condition without any broader phenotype. In some training environments, autism awareness and best clinical practices are simply lagging behind. In high-functioning individuals, AT are often masked by co-morbidities like anxiety and mistaken for other conditions, e.g., social phobia, obsessive-compulsive disorder, ADHD, narcissism, depression, and eating disorders [18]. Women with ASD may be further under-recognized due to better imitation and learning of neurotypical behaviour, referred to as a “camouflage” phenomenon [28].

Moreover, many characteristics associated with AT are normalized and valued in medicine: attention to detail, emotional detachment, “passion” for a certain topic, and “expertise” in a narrow field. Interestingly, significant overlaps have been described between high-functioning autism, ADHD, and giftedness [19], defined as an IQ above 130, prevalent among physicians.

Performance metrics in medicine certainly contribute to normalizing AT. Traditionally, knowledge has been more valued than attitudes and communication [29]. High academic achievement has often excused poor social skills. While central to medical admissions and recruitment processes, it captures knowledge acquisition but is an unreliable indicator of social communication skills [30, 31]. In training, with attitudes more difficult to assess, programs have often relied on quantitative assessment tools focusing on knowledge and memory.

Finally, mental health issues in medical students and physicians remain taboo. As in the general population, learners and doctors with ASD are likely to have a late diagnosis, if ever confirmed [1]. Lack of insight, wrong self-diagnosis, fear of stigma, and doubt in the benefit of engaging in this process can hinder diagnosis.

## For better educational and health outcomes

Why is recognizing AT increasingly important in medical education? We believe that social communication skills can improve to a certain extent. Learning potential can be optimized by investing in comprehensive educational approaches, the sooner the better especially with struggling learners displaying AT. Lack of insight can be a major hurdle, and early attention to metacognitive skills can prove helpful. Failure to recognize AT-related gaps and vulnerabilities in competence development can have negative consequences, including on patients’ health. Awareness of AT encourages educators to rely more on describing learners’ observable behaviours and to render the implicit more explicit. Where expected competencies are achieved, documentation of effective coping strategies can support lifelong learning. Otherwise, acknowledgement of limitations may allow meaningful reframing of difficulties and effective career counselling adapted to personal strengths.

Ultimately, effective social communication for all involved in healthcare is crucial. Social expectations for doctors go beyond medical expertise and the delivery of safe quality care relies on constructive teamwork. Competency-based medical education frameworks such as CanMEDS [32], ACGME core competencies [33], and CFPC assessment objectives [34] integrate communication, collaborative skills, and professionalism across all medical disciplines.

In rapidly evolving healthcare environments, previously acceptable behaviours may become overt deficits as the gap grows between expected competences and an individual’s adaptability. Interprofessional collaboration may make individual deficits evolve into a broader organizational dysfunction. Once in practice, physicians’ status may impede patients and peers from expressing genuine feedback, limiting opportunities to learn, motivation to improve, and emotional self-regulation to occur. Systemic gaps in healthcare for those soft but essential skills may persist over generations.

## Future directions

As AT are respectfully and meaningfully addressed as part of neurodiversity in society, in workplaces, and in higher education [35], medicine also needs to engage in this movement. Despite the risks of stigma and prejudice associated with this proposal, we wish to underline the feasibility and the necessity of conciliating diversity, inclusivity, and dignity with accountable standards for medical competence. The risks of not engaging in this conversation must also be recognized.

We believe the solution rests in focusing on optimizing outcomes. Everyone has potential for improvement until proven otherwise, regardless of a formal diagnosis of ASD or signs of AT. Although the latter may explain some learning problems, it must not lower the bar for expected competencies in future physicians, including robust self-regulating skills.

This perspective provides a starting point for a necessary new conversation in medical education. AT need to be considered as a factor when confronted with learning or interpersonal challenges in medicine. A “neurodiversity lens” that acknowledges AT should be part of comprehensive learning and teaching strategies, to optimize everyone’s potential in training and practice, regardless of how neurotypical or neuroatypical they are. This lens can ease tensions in situations of challenging interactions and enable more constructive educational dialogues.

Next steps might include guidance for others offered by colleagues with AT who have successfully overcome their learning challenges. Furthermore, we should answer important questions, such as: “What should I do, as an educator, if I recognize signs of AT in a learner?”, “What strategies best support improvement of this specific skill?”, “How can I, as a learner, better understand situations that I do not entirely see?”, and “How do we know if we have reached the best possible outcome?”

Revisiting our professional stories through a neurodiversity lens may nurture a better understanding of AT. Some readers may recognize these traits in their learners, colleagues, patients and even in themselves or their entourage. Emotional intelligence and collective wisdom are indissociable pieces of this delicate conversation. With much yet to be explored and understood, any future directions in research, teaching, and leadership in medicine must prospectively aim at improving educational and health outcomes while nurturing diversity with respect and dignity.
